# In vivo characterization of target cells for acute elephant endotheliotropic herpesvirus (EEHV) infection in Asian elephants (*Elephas maximus*)

**DOI:** 10.1038/s41598-020-68413-4

**Published:** 2020-07-09

**Authors:** Thunyamas Guntawang, Tidaratt Sittisak, Saralee Srivorakul, Varankpicha Kochagul, Kornravee Photichai, Chatchote Thitaram, Nattawooti Sthitmatee, Wei-Li Hsu, Kidsadagon Pringproa

**Affiliations:** 1 Department of Veterinary Biosciences and Veterinary Public Health, Faculty of Veterinary Medicine, Chiang Mai University, Chiang Mai, 50100 Thailand; 2 Veterinary Diagnostic Laboratory, Faculty of Veterinary Medicine, Chiang Mai University, Chiang Mai, 50100 Thailand; 3 Center of Excellence in Elephant and Wildlife Research, Chiang Mai University, Chiang Mai, 50100 Thailand; 4 Department of Companion Animals and Wildlife Clinics, Faculty of Veterinary Medicine, Chiang Mai University, Chiang Mai, 50100 Thailand; 5 Graduate Institute of Microbiology and Public Health, College of Veterinary Medicine, National Chung Hsing University, Taichung, 402 Taiwan

**Keywords:** Infection, Herpes virus, Viral reservoirs

## Abstract

Elephant endotheliotropic herpesvirus-hemorrhagic disease (EEHV-HD) is a dangerous viral infectious disease in young Asian elephants. Despite hypotheses underlying pathogenesis of the disease, it is unclear which cell types the virus targets during acute or persistent infections. This study investigated the tissues and target cells permissive for EEHV infection and replication in vivo. Rabbit polyclonal antibodies against the non-structural proteins of EEHV, DNA polymerase (EEHV DNAPol), were generated and validated. These were used to examine EEHV infection and replication in various tissues of acute EEHV-HD cases and compared to an EEHV-negative control. The results indicated that viral antigens were distributed throughout the epithelia of the alimentary tract and salivary glands, endothelia and smooth muscle cells, and monocytic lineage cells of the EEHV-infected elephants. Moreover, EEHV DNAPol proteins were also found in the bone marrow cells of the EEHV1A-HD and EEHV1A/4-HD cases. This study demonstrated for the first time the target cells that favor in vivo EEHV replication during acute infection, providing a promising foundation for investigating EEHV propagation in vitro.

## Introduction

Elephant endotheliotropic herpesvirus-hemorrhagic disease (EEHV-HD), caused by EEHV, is a dangerous viral hemorrhagic disease affecting Asian elephants (*Elephas maximus*) worldwide^1–3^. Clinically, the disease causes facial swelling, tongue cyanosis, acute onset of thrombocytopenia, monocytopenia and hemorrhaging in the visceral organs; this can be fatal, especially in elephants younger than 8 years old^1,4,5^. EEHV (genus *Proboscivirus*, family *Herpesviridae*, subfamily *Betaherpesvirinae*) is an enveloped, linear double-stranded DNA virus that has been classified into eight genotypes: EEHV1A, 1B and EEHV2-7^1,6^. The genomes of genotypes EEHV1A, 1B and 4 have been completely sequenced; their nucleotides range from ~ 180 to ~ 206 kb^7–9^. EEHV1A and EEHV1B have protein encoding segments of 116 and 115 open reading frames (ORFs), respectively^9^, while EEHV4 has 119 ORFs^7,8^. EEHV1A has 37 conserved core genes that are similar to other herpesviruses; 15 genes that are similar to either both betaherpesviruses and gammaherpesviruses or just betaherpesviruses; 3 genes that are lost from the betaherpesviruses; and 60 new genes absent from all herpesviruses^8^. Despite this extensive analysis of EEHV’s genome, much of what underlies EEHV-HD remains uncertain or controversial, including which tissues and cell types are permissive for EEHV infection and replication.

Infection and replication of *Betaherpesviruses*, such as Human cytomegaloviruses (HCMV), requires several sequential steps of progeny virion production^10,11^. HCMV infects a broad range of target tissues in humans, including epithelia of the oropharynx, endothelia, fibroblasts and resident macrophages^11–16^; infection begins with the virus attaching to host cell receptors, including epidermal growth factor receptor (EGFR), platelet-derived growth factor receptor alpha (PGFRα), or integrin^17–19^. Then, the virus proceeds to replicate its own DNA by producing DNA polymerase, the non-structural protein required for DNA prolongation^11^. The presence of viral DNA polymerase is, thus, indicative of viral nucleic acid replication, and has been shown to be a marker for viral infection and replication in several types of viruses^20,21^.

Antibodies against the envelope glycoprotein B (gB) of EEHV have been generated and used to determine EEHV gB antigens in elephant tissues, including the salivary glands, gastrointestinal epithelia and peripheral blood monocytes^22^. However, these antibodies, derived from structural proteins, are not appropriate as indicators of viral replication, since the presence of EEHV gB in the cytoplasm of monocytes/macrophages could be due to their phagocytic activity^23^. Antibodies derived from non-structural proteins offer several advantages, yet to the best of our knowledge, none have been developed to study EEHV. Thus, this study aimed to develop antibodies against the non-structural proteins of EEHV (i.e. DNA polymerase); and then use these to investigate the target cells for EEHV replication in fatal cases of EEHV-HD in Asian elephants.

## Results

### Production and characterization of polyclonal antibodies against EEHV DNAPol proteins

**Figure 1:**
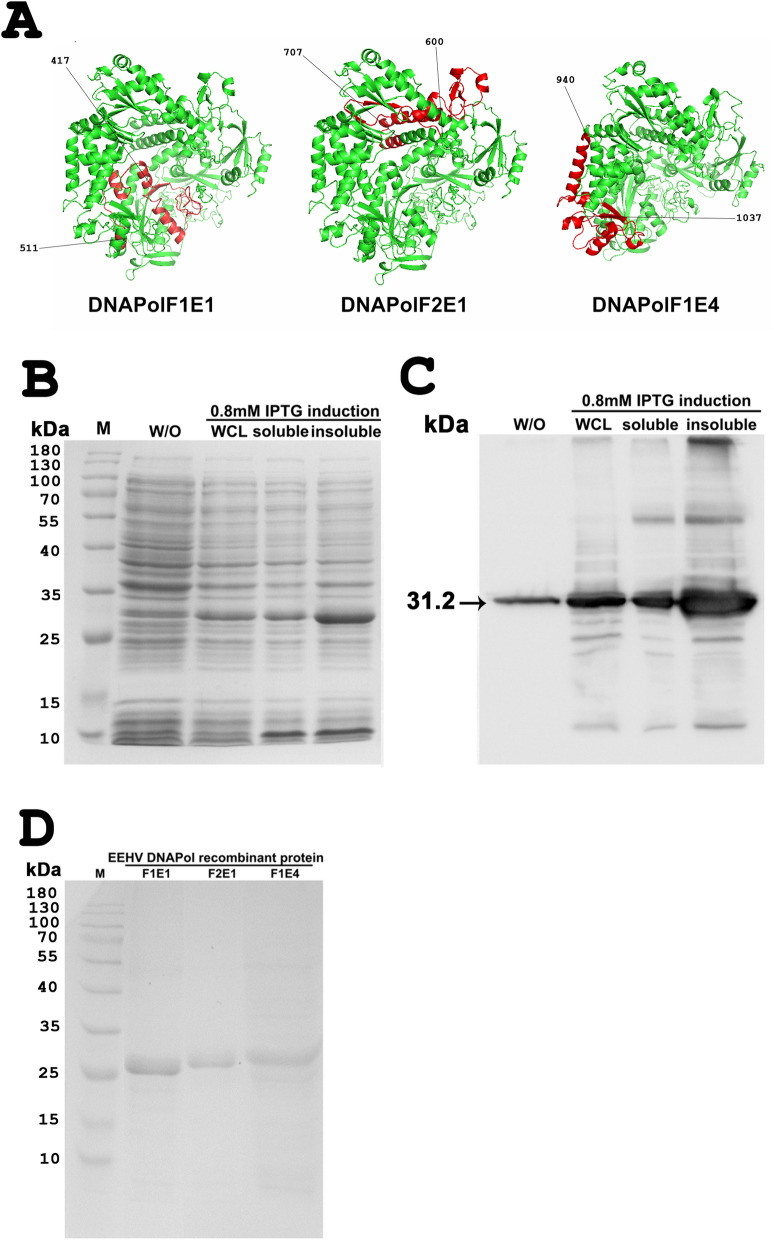
Three dimensional (3D) protein structures and characterization of the EEHV DNAPol recombinant proteins. Predicted regions of DNAPolF1E1, DNAPolF2E1 and DNAPolF1E4 were obtained from the U38 at the amino acid position 417–511 and 600–707 of EEHV1A and 940–1,037 of EEHV4, respectively (**A**). SDS-PAGE (**B**) and western blot analysis (**C**) of DNAPolF2E1 from the BL-21 *E. coli* induced by 0.8 mM IPTG showed a molecular size of 31.2 kDa, which mainly produced in insoluble form (**C**). M: Marker, W/O: cells without induction, WCL: Whole cell lysate after induced with 0.8 mM IPTG. SDS-PAGE analysis of the purified EEHV DNAPol histidine-tagged proteins through Ni–NTA column indicated molecular protein sizes of DNAPolF1E1, DNAPolF2E1 and DNAPolF1E4 of 29.9, 31.2 and 30.6 kDa, respectively (**D**). The 3D structures were generated from digital information available at I-TASSER server and image was modified with Adobe Photoshop CS6 v.13.0.1.

To generate the polyclonal antibodies against the EEHV non-structural proteins, two recombinant protein fragments of U38 (DNA polymerase) from EEHV1A (DNAPolF1E1 and DNAPolF2E1), and one fragment of U38 from EEHV4 (DNAPolF1E4) were generated. The predicted structures from the I-TASSER software revealed that EEHV1A DNAPol had a template modeling (TM) score of 0.80 ± 0.09, while EEHV4 DNAPol had a TM score of 0.74 ± 0.11, which was similar to the structure of herpes simplex virus-1 (HSV-1) DNA polymerase (data not shown). Predicted regions of DNAPolF1E1, DNAPolF2E1 and DNAPolF1E4 were at positions 417–511 and 600–707 for EEHV1A and 940–1,037 for EEHV4, respectively (Fig. 1A).

SDS-PAGE and western blot analysis showed that candidate EEHV DNAPol proteins were induced by IPTG (0.8 mM) treatment; they were present in the insoluble form (Fig. 1B,C). By SDS-PAGE analysis indicated the histidine-tagged DNAPol proteins, the approximate protein sizes of DNAPolF1E1, DNAPolF2E1 and DNAPolF1E4 were 29.9, 31.2 and 30.6 kDa, respectively (Fig. 1D). To characterize whether the rabbit antisera used for further immunohistochemical studies is targeting the immunogen, recombinant protein was SDS-PAGE, immunoblotted with the rabbit anti-EEHV DNAPol antibodies and showed the molecular weight size of DNAPolF2E1 at 31.2 kDa (Fig. S1). Demonstration of EEHV antigens in tissue lysate of the EEHV1A-infected case by western immunoblotting indicated the protein weight size of the EEHV DNA polymerase at ~ 117 kDa (Fig. S2).

### Localization of the EEHV DNAPol proteins in the EEHV-HD cases

**Figure 2:**
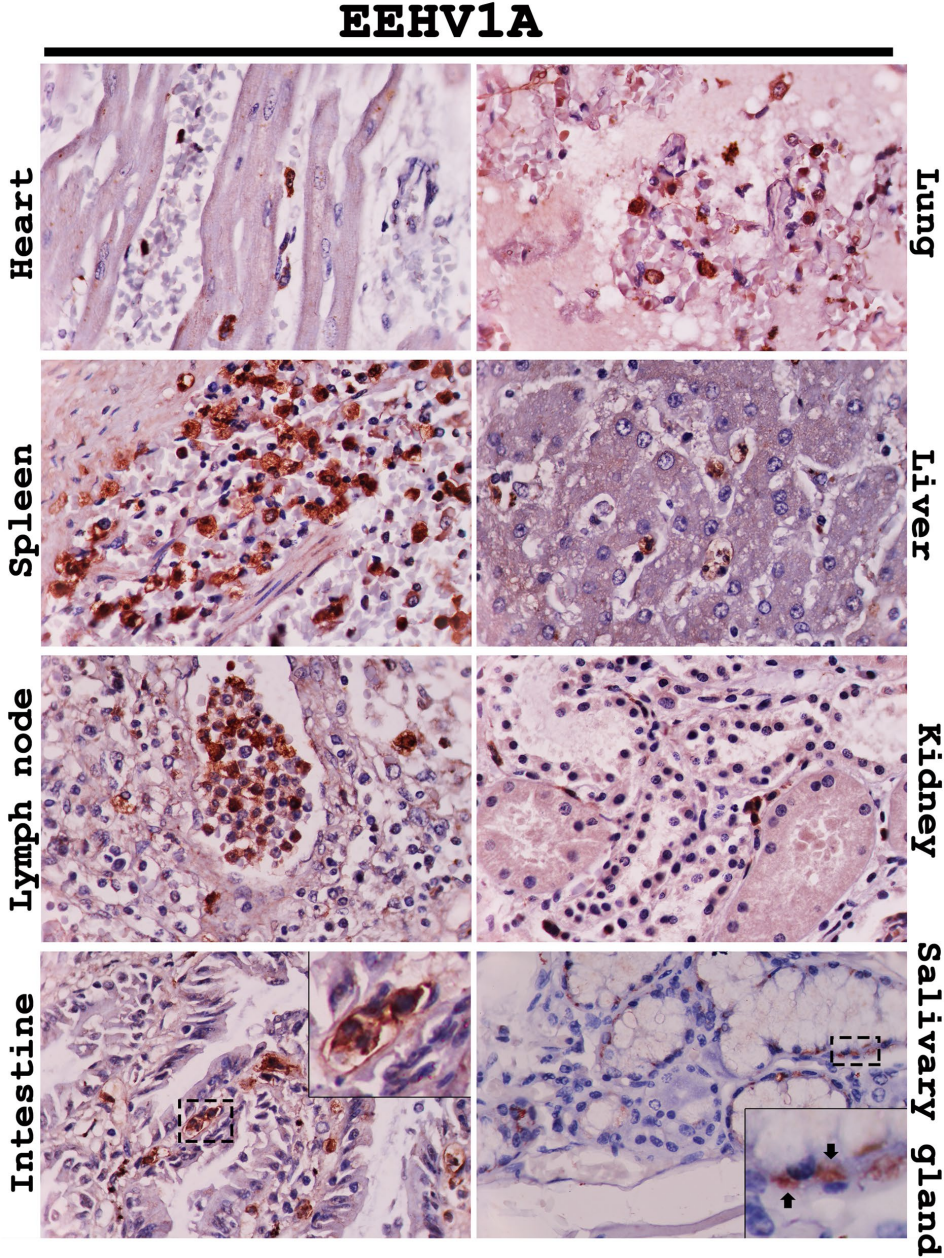
Representative photomicrographs of the EEHV DNAPol proteins in the EEHV1A-HD cases by immunohistochemistry. Immunolabeling positive cells for EEHV DNAPol were observed in the monocytes/macrophages (intestine, inset) of the heart, lung, spleen, liver, lymph node, kidney and intestine. Epithelial cells of the salivary gland were shown to be immunolabeling positive in the cytoplasm of the infected cells (inset, arrow). Image was modified with Adobe Photoshop CS6 v.13.0.1.

**Figure 3:**
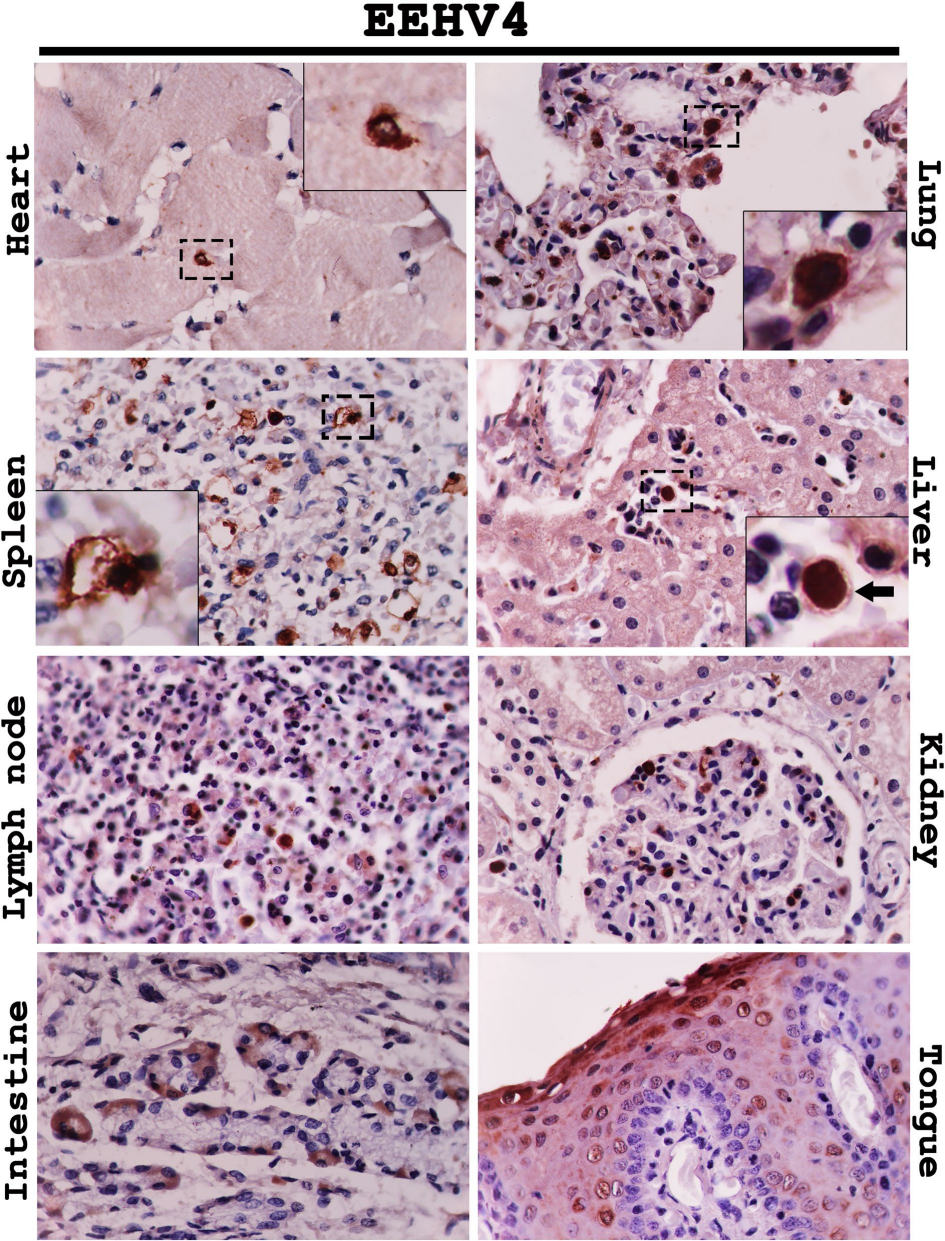
Representative photomicrographs of the EEHV DNAPol proteins in the EEHV4-HD cases by immunohistochemistry. Detection of the EEHV DNAPol proteins in the EEHV4-HD calves showed immunolabeling for EEHV DNAPol in the monocytes/macrophages (inset) of the heart, lung, spleen, lymph node and kidney, while immunolabeling positive cells were seen in the endothelia of hepatic sinusoid (inset, arrow), crypt epithelia of the intestine and squamous epithelia of the tongue. Image was modified with Adobe Photoshop CS6 v.13.0.1.

**Figure 4:**
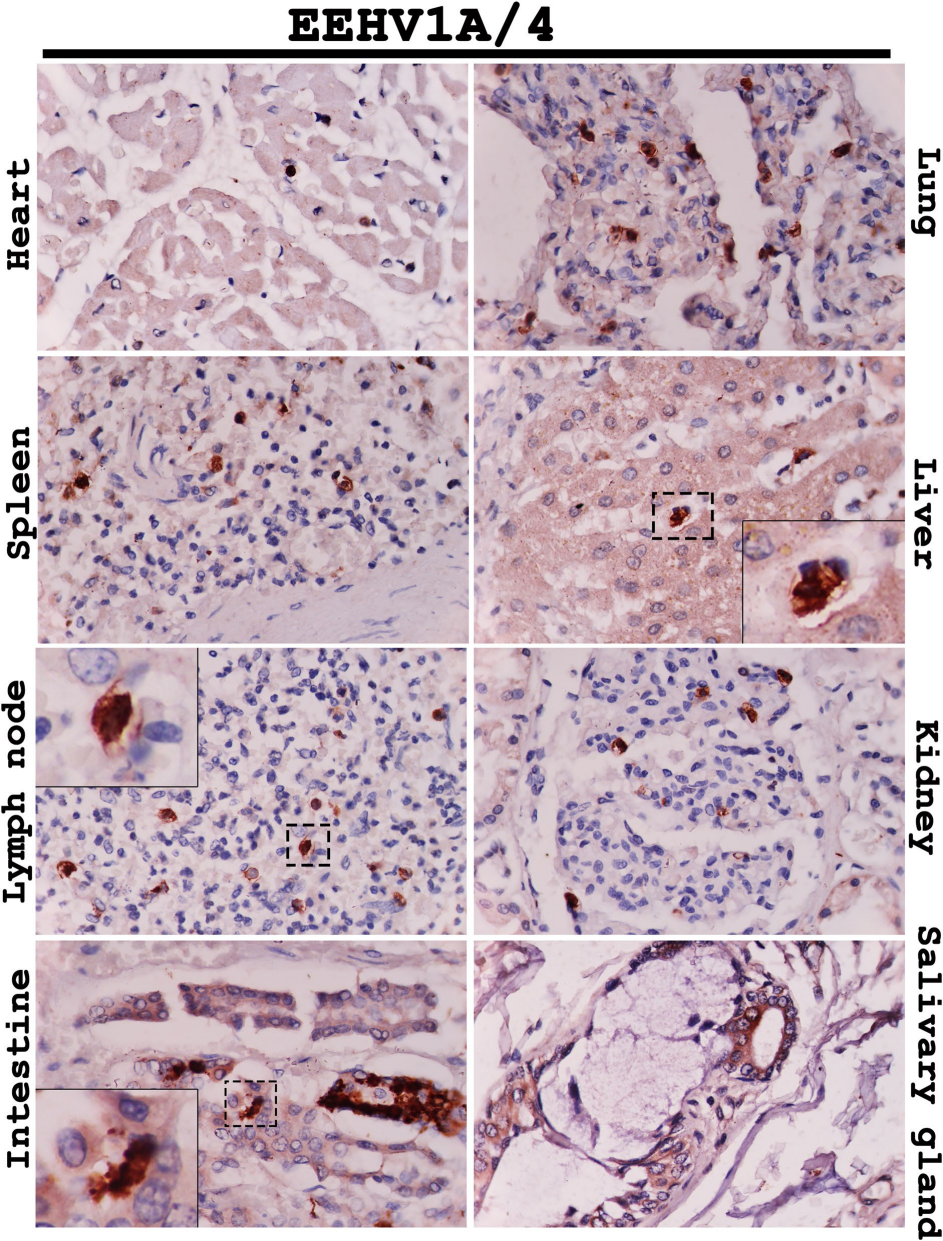
Representative photomicrographs of the EEHV DNAPol proteins in the EEHV1A/4-HD cases by immunohistochemistry. EEHV DNAPol proteins were observed in the monocytes/macrophages (inset) of the heart, lung, spleen, liver, lymph node and kidney. Epithelial cells of the intestinal crypts (inset) and salivary gland were also shown to immunolabel positive for the EEHV DNAPol antibodies. Image was modified with Adobe Photoshop CS6 v.13.0.1.

Immunohistochemical labeling for the EEHV DNAPol proteins of the EEHV1A-HD, EEHV4-HD, and EEHV1A/4-HD cases revealed that the EEHV DNAPol antigens were distributed in a variety of tissues (Table 1). The major target cells of EEHV were monocytes in blood vessels and macrophages of various internal organs, including the heart, lung, spleen, liver, kidney and lymph nodes (Figs. 2, 3, 4). Moreover, EEHV DNAPol immunolabeling positive cells were also observed in the endothelia and smooth muscle cells of small blood vessels, and epithelia of the trunk, tongue, salivary glands and gastrointestinal tract of the EEHV-HD cases (Figs. 2, 3, 4). It should be noted that no staining was observed in either sections of the EEHV-negative case probed with the rabbit anti-EEHV DNAPol antibodies (Fig. S3), or the EEHV-HD tissues probed with rabbit pre-immunized sera (Fig. S4). These findings indicated the specificity of the rabbit anti-EEHV DNAPol antibodies for the EEHV antigens.

**Table 1:** Distribution of EEHV DNAPol antigens in the EEHV-HD cases determined by immunohistochemistry.

Organs^b^	Genotypes^a^		
	EEHV1A	EEHV4	EEHV1A/4
Heart	+ + +	+ +	+
Lung	+ + +	+ + +	+ +
Spleen	+ + +	+ + +	+ +
Liver	+ +	+ + +	+
Lymph node^c^	+ + +	+ +	+ +
Kidney	+ +	+ + +	+
Pancreas	+	+	+
Stomach	+	+ +	+
Intestine^d^	+ + +	+ + +	+ + +
Tonsil	NA	+ +	NA
Trunk	+ +	NA	NA
Tongue/salivary gland	+ +	+ + +	+ + +
Spinal cord	+	+	+
Thyroid gland	NA	+	NA
Nerve ganglion	NA	+	NA
Aorta	+	+	NA
Thymus	NA	+	NA
Bone marrow	+ + +	NA	+ +

^a^Occurrence and intensity of signal: “ + ” = mild, “ + + ” = moderate, “ + + + ” = high.

^b^Cells determined, including endothelia, blood leukocytes, macrophages, epithelia.

^c^At least two locations (inguinal, mandibular or mesenteric lymph nodes) were determined.

^d^Small and large intestines, NA: not available.

**Figure 5:**
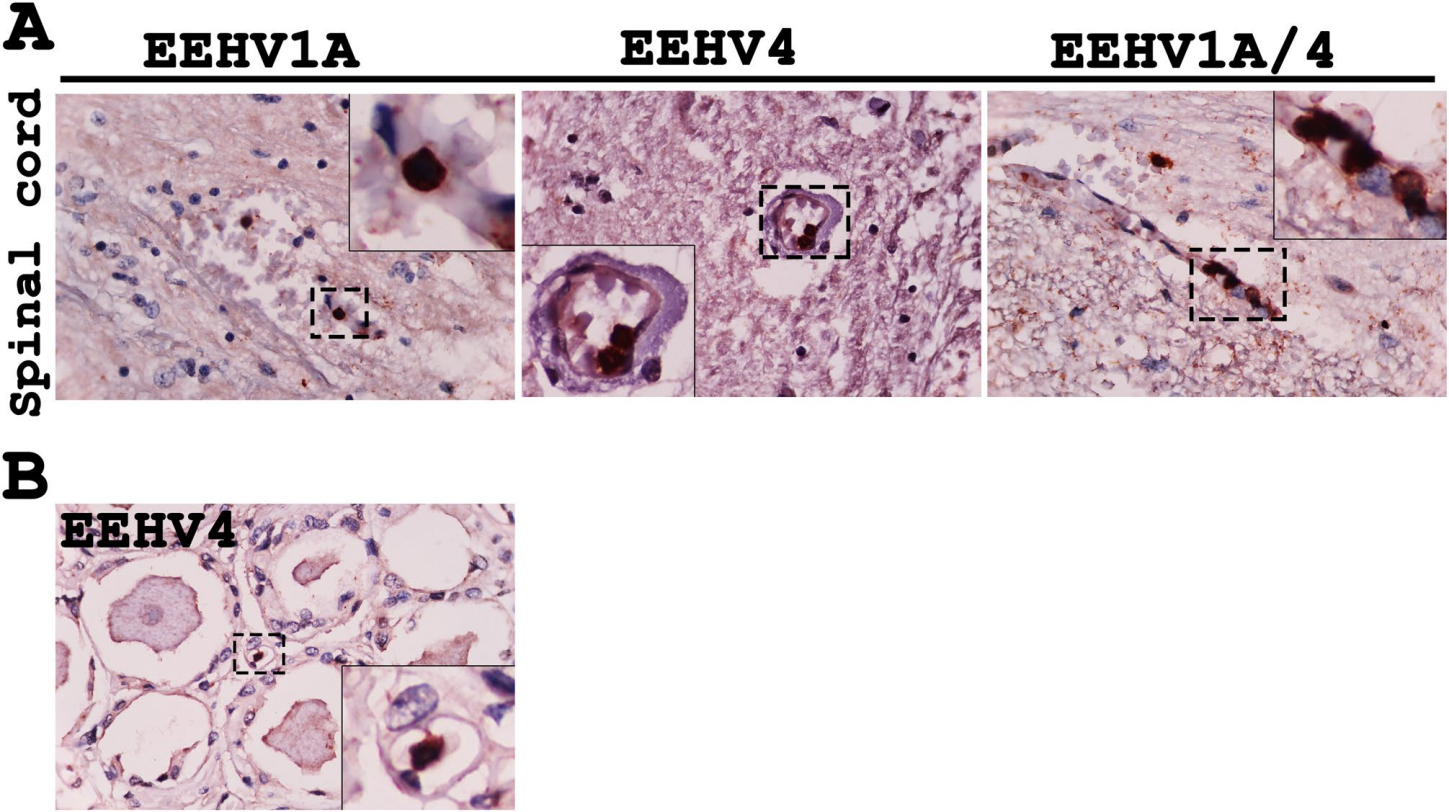
Immunohistochemical labeling for the EEHV DNAPol in the central nervous system (CNS) and peripheral nervous system (PNS). Spinal cords of the EEHV1A-HD, EEHV4-HD and EEHV1A/4-HD cases were immunostained with rabbit anti-EEHV DNAPol antibodies and indicated that only the infiltrating blood leukocytes were positive (**A**; inset). Nerve ganglion of the EEHV4-HD case also showed the immunolabeling positive cells of the leukocyte within blood vessels (**B**; inset). Image was modified with Adobe Photoshop CS6 v.13.0.1.

EEHV DNAPol immunolabeling positive cells in the central nervous system (CNS) and peripheral nervous system (PNS) were distributed in the infiltrating blood leukocytes (Fig. 5).

### EEHVs preferentially infected small- to medium-sized blood vessels

**Figure 6:**
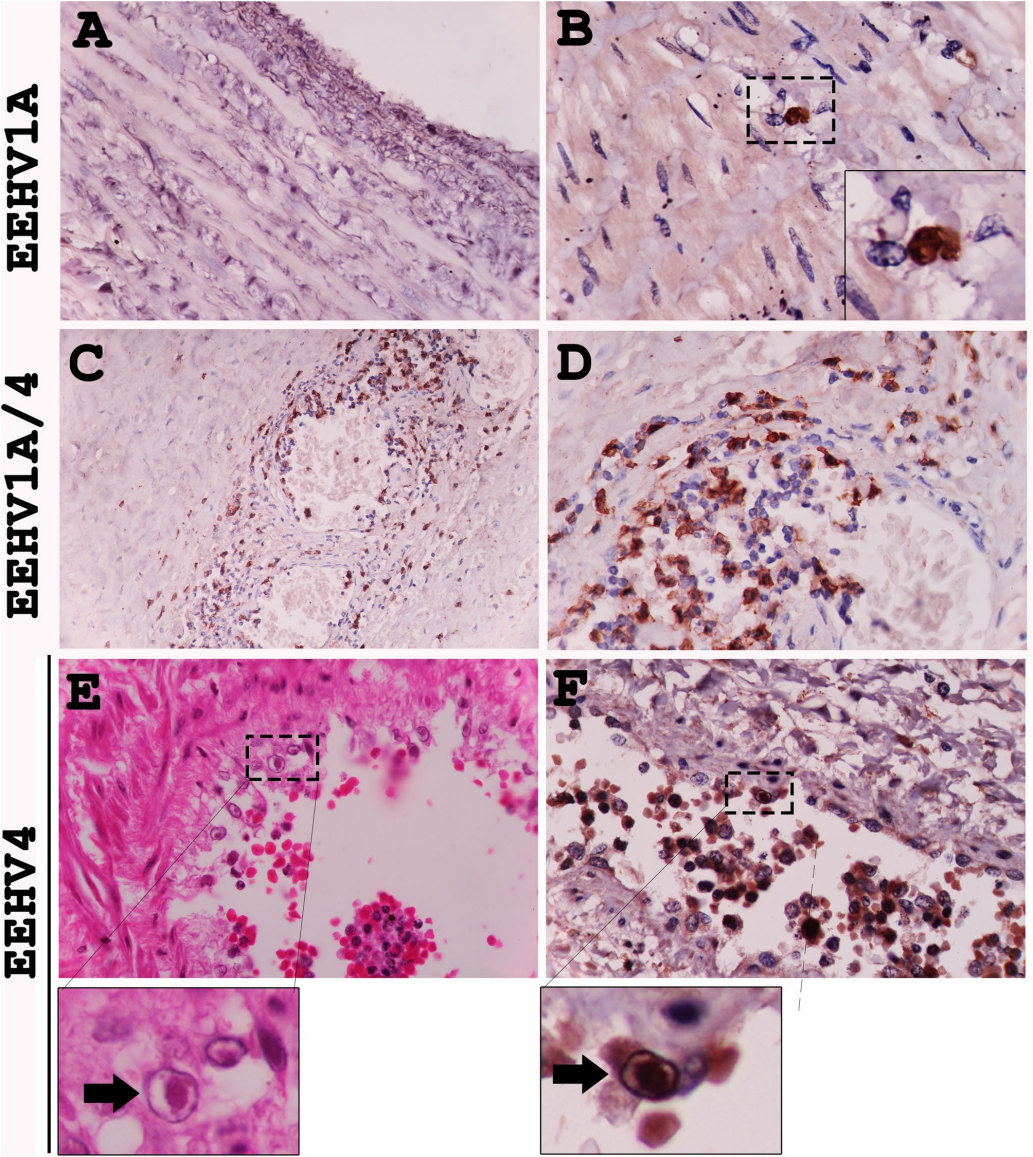
EEHV preferentially infect certain types of endothelia of the visceral organs. Immunohistochemical staining of the EEHV-HD calves with polyclonal anti-EEHV DNAPol antibodies revealed that endothelia and smooth muscle of the large blood vessel (aorta; **A**) were not targeted by EEHV, while infiltrating monocytes in the vasa vasorum of tunica adventitia were shown to be infected by EEHV (**B**). On the other hand, EEHV are prone to infect and replicate in the small blood vessels (artery and vein) of the internal organs (**C**–**F**). Basophilic intranuclear inclusion bodies (arrows) of the EEHV were observed in the endothelia of small blood vessel (**E**, inset; H&E) and positive for EEHV DNAPol immunolabeling (**F**, inset). Image was modified with Adobe Photoshop CS6 v.13.0.1.

Our results indicated that EEHV preferentially infected and replicated in the endothelia and smooth muscle cells of the small blood vessels and microvessels, including arterioles, venules and capillaries; EEHV did not target the endothelia and smooth muscle cells of large blood vessels (Fig. 6A). However, EEHV DNAPol antigens were observed in the infiltrating monocytes in the vasa vasorum at the tunica adventitia of the aorta (Fig. 6B). Microscopically, it could be seen that EEHV infected the endothelia by infiltrating the inflammatory cells in the tunica media and tunica intima, with sloughing of the necrotic endothelia (Fig. 6C,D). The EEHV-infected endothelia presented with large basophilic intranuclear inclusion bodies, as observed by histopathological (Fig. 6E, arrow) and immunohistochemical labeling with EEHV DANPol antibodies (Fig. 6F, arrow).

### EEHV targeted elephant bone marrow cells

**Figure 7:**
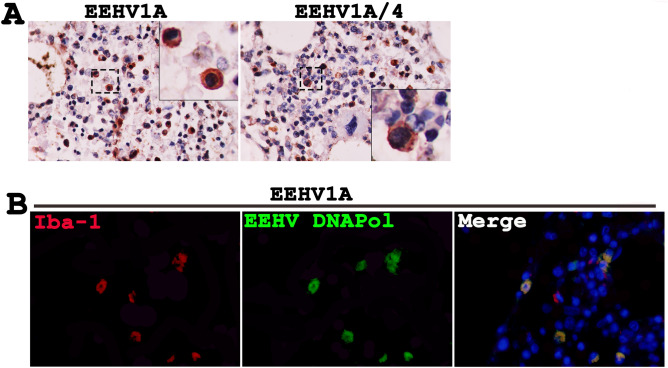
Bone marrow cells of the elephant calves were targeted by the EEHV, as determined by immunohistochemistry and double immunofluorescence. Bone marrow of the EEHV1A-HD and EEHV1A/4-HD calves were immunostained with anti-EEHV DNAPol antibodies and showed positive in the cytoplasm and nucleus of the Iba-1 positive cells (**A**, **B**), indicating their monocytic phenotypes. Image was modified with Adobe Photoshop CS6 v.13.0.1.

To investigate whether EEHV targeted bone marrow cells, bone marrow from the EEHV1A-HD and EEHV1A/4-HD calves were immunostained to determine the presence of EEHV DNAPol proteins. The results found immunolabeling positive cells in the different types of bone marrow cells (Fig. 7A), including Iba-1 positive cells (Fig. 7B). Double immunolabeling of the Iba-1 and EEHV DNAPol antibodies revealed that more than 70% of the Iba-1 positive cells were targeted by the EEHV (Fig. 7B). However, EEHV DANPol immunolabeling positive cells were also found in other cell types (Fig. 7B).

## Discussion

Our study demonstrated for the first time that EEHV targeted a broad range of cells in young Asian elephants during acute infection. As EEHV infection in elephants induces acute onset with clinical signs and lesions mostly involving the cardiovascular system, EEHV is primarily acknowledged as an endotheliotropic virus^3,24,25^. Richman et al. hypothesized that vascular endothelial cells are permissive for EEHV infection and might play a major role underlying virus dissemination and pathogenesis of EEHV-HD^3^. Moreover, our previous report using an in situ hybridization test demonstrated that not only endothelia, but also monocytes/macrophages harbored EEHV genomes^26^. However, the presence of viral DNA in a cell body is not enough to show whether those cells were targeted for virus replication, as it is not determinative of whether viral proteins were translated there.

Our study’s findings reaffirmed^1,3,26^, that EEHV replicated in the vascular endothelia of the infected Asian elephants. Furthermore, the present study indicated that EEHV seems to preferentially infect only certain types of endothelia, including small arteries and veins, arteriole, venules and capillaries, similar to that observed in HCMV infection in humans^27^. Endothelial cells appear to play an important role in the maintenance of viremia for certain viruses^10^. Although we do not know yet why EEHV only targets the endothelia of small blood vessels, these cells are known for their heterogeneous populations and expression of unique cell surface antigens, as well as having receptors for only specific pathogens^27–29^. In other herpesviruses, it has been shown that only endothelial cells from small vessels or capillaries harbored murine cytomegalovirus (MCMV) and that these were the latency sites for persistent MCMV infection^30^. Thus, our findings that EEHV targeted the small blood vessels for replication strongly suggest that these blood vessels may express unique cell surface antigens required for EEHV, and thus, play a role in pathogenesis of EEHV-HD. It is also possible that higher pressure in large blood vessels^31^ keeps the virus-infected leukocytes from adhering to the endothelial cells there, while they more easily adhere to the endothelia of small blood vessels.

EEHV infection and replication in the epithelial cells of the oropharynx, salivary glands, and intestines of elephants likely reflects the role these cell types play in horizontal transmission of the virus. Our current study supports our prior hypothesis^22^ that EEHV is transmitted via saliva and intestinal excretion. In betaherpesviruses, such as HCMV, the virus is transmitted mainly by bodily secretions, including breast milk, saliva, urine, and genital excretion^13^. To date, only saliva and intestinal secretions have been demonstrated or speculated as the major routes of EEHV transmission^22,32,33^; whether any of the other bodily secretions are involved has not been determined. Moreover, given our findings that epithelia of the trunk and tongue are permissive for EEHV replication, it is reasonable to speculate that the primary infection site of EEHV in recipient animals is the epithelia of the trunk or oral cavity; and from there the virus spreads to other target organs via circulating blood leukocytes.

Our present findings are consistent with our previous conclusion that monocytes may serve as carrier cells for viral dissemination in EEHV-HD^23^. Infection and replication of EEHV in endothelial cells, monocytes and macrophages suggested that these cell types may facilitate systemic spread and are directly involved in the magnitude of viremia in the infected host. Our previous findings hypothesized that acute or sporadic monocytopenia observed in EEHV-infected cases is due to both increased apoptosis of monocytes/macrophages and extravasation of the peripheral blood monocytes through blood vessels^23^. This seems reasonable since a significant increase of TUNEL positive cells and Iba-1 positive cells was observed in tissue parenchyma of the EEHV-infected internal organs^23^. However, based on results in the present study and that EEHV gB was found in the monocytes/macrophages^22^, and that EEHV DNA loads in the infected cases were observed in the PBMCs rather than serum^25,32^, it is strongly suggested that monocytes/macrophages are, in fact, major targets for EEHV replication in vivo. Evidence has shown that the violence of viremia in several models of viral infections, including African swine fever virus, dengue virus and HIV, is facilitated by infection of blood leukocytes^10^. These viruses infect and replicate in monocytes/macrophages, which amplifies rather than reduces viremia^10^. Betaherpesviruses, such as porcine CMV, MCMV and HCMV, have also been shown to target and replicate in monocytes/macrophages^34–36^. This lends weight to our conclusion that monocytes/macrophages serve as major sites of EEHV replication.

The present study has demonstrated for the first time that bone marrow cells of the EEHV-infected cases are also targeted by EEHV during acute infection. This finding leads to the possibility that bone marrow cells may serve as a reservoir for EEHV during persistent infection, as seen in other betaherpesviruses^13,15^, although the presumed persistent forms of EEHV in elephants have not been identified yet^6^. In summary, the present study demonstrated the permissive cell types required for acute EEHV infection and replication in vivo. The broad target cell range of EEHV might significantly influence the pathogenesis of the virus in Asian elephants and will be useful for further in vitro EEHV studies.

## Methods

### Animal samples and ethical statement

Archives of formalin-fixed, paraffin-embedded (FFPE) tissue from elephants with EEHV1A-HD (n = 3), EEHV4-HD (n = 2), and EEHV1A/4-HD (n = 2) were included in the study. Tissue (n = 1) from an elephant calf that died from causes unrelated to an EEHV infection, as determined by polymerase chain reaction (PCR), served as the negative control. Details of the selected cases are summarized in Table 2.

**Table 2:** Summary of animals’ history and specimens recruited in this study.

Animals no	Sex	Age	EEHV genotype	Test performed for EEHV DNAPol products	Remark (ref.)
1	Female	2 years	EEHV1A	IHC, IFA	^22,26^
2	Female	3 years	EEHV1A	IHC	^22^
3	Male	2 years	EEHV1A	IHC, IFA, WB	Acute death in 2 days after showing EEHV-HD clinical signs in June 2019
4	Female	2 years	EEHV4	IHC, IFA	^23,26^
5	Female	5 years	EEHV4	IHC	^22,23^
6	Female	6 years	EEHV1A/4	IHC, IFA	^23^
7	Female	4 years	EEHV1A/4	IHC	^23^
8	Male	Stillbirth	Negative	IHC, IFA	^22,23,26^

IHC: immunohistochemistry; IFA: immunofluorescence; WB: western blot.

Seven New Zealand White rabbits, 6–8 weeks old, were used to produce antibodies. All methods were performed in accordance with the relevant guidelines and regulations. Animal experiment protocols were approved by the Institutional Animal Care and Use Committee, Faculty of Veterinary Medicine, Chiang Mai University, Chiang Mai, Thailand (approval number: FVMACUC. R11/2561). Genetically modified experiments and recombinant protein production were performed in the appropriate bio-containments of the biosafety level 2 (BSL2) laboratories.

### Polymerase chain reaction (PCR) and gene sequencing

The total DNA from frozen tissues obtained from the heart, spleen and lymph nodes of EEHV-HD calves and an EEHV-negative control calf was extracted using a commercial DNA extraction kit (Machery-Nagel GmbH, Dauren, Germany). To determine the EEHV-infection status, a PCR test for the polymerase and terminase genes of EEHV were performed, as previously described^3^. Thereafter, the positive PCR samples of EEHV1A and EEHV4 were used as templates for generating the recombinant DNAPol proteins using different primer pairs. The sequences of EEHV DNA polymerase proteins were choosing based on their hydrophilicity, negative score region and the high antigenicity sites by using the computer software, as described below.

### Generation and production of recombinant EEHV DNAPol proteins

Primers of DNA polymerase (DNAPol) genes (Table 3) of EEHV1A (accession no. AGG16071.1) and EEHV4 (accession no. ALM25982.1) with the restriction enzyme cutting sites were generated and obtained using the Kyte & Doolittle scale (https://web.expasy.org/protscale/), I-TASSER (Iterative Threading ASSEmbly Refinement) server version 5.1^37–39^ and SnapGene software version 1.1.3. Conventional PCR was performed under the following conditions: 98 °C for 2 min; 35 cycles of 98 °C for 10 s, 60 °C for 30 s, and 72 °C for 20 s; and a final extension at 72 °C for 7 min. The PCR products were analyzed by electrophoresis and specific bands were observed under a UV illuminator. The PCR products corresponding to the expected genome size were then further analyzed by DNA sequencing.

**Table 3:** Primer list for generating the recombinant EEHV DNAPol proteins.

Name	Sequence	Restriction enzyme	Protein size (kDa) (including TrxA)	Amino acid position (AA)
DNAPolF1E1	5′-gggGGATCCCccctacgtgatagggcgatg-3′ 5′-ggggCTCGAGgtcgtctcttctaaacaaaactgggatct-3′	BamHI XhoI	29.9	417–511
DNAPolF2E1	5′-ggggGGATCCgactggattctacaatactccggtg-3′ 5′-ggggCTCGAGcgttagttttagagccagttgttgtttgtc-3′	BamHI XhoI	31.2	600–707
DNAPolF1E4	5′-ccgAAAGCTTttcttcaacctcgtggcgcttatc-3′ 5′-ggggCTCGAGgttgttgttccgggcgta-3′	HindIII XhoI	30.6	940–1,037

The PCR products containing the restriction sites were inserted into the pET32b + vector (Novagen, Merck, Darmstadt, Germany) and transformed into Top 10 *Escherichia coli* (Novagen, Merck, Darmstadt, Germany). Thereafter, the plasmid of positive colonies, as verified by PCR and DNA sequencing, were extracted and inserted into the BL-21 *E. coli* for protein expression, as previously described^40^. Briefly, the BL21 *E. coli* colonies were picked and inoculated in Luria–Bertani (LB) broth containing 100 µg/mL ampicillin and shook at 37 °C until the OD_600_ increased to 0.6–0.8. Then, protein expression was induced by adding 0.8 mM isopropyl β-d thiogalactopyranoside (IPTG) at 37 °C for 5 h. Bacteria were collected by centrifugation at 6,000 rpm, 4 °C for 15 min. After discarding the supernatant, cells were lysed in binding buffer containing 0.05 M Tris–HCl, 10 mM imidazole, 0.5 M NaCl and 6 M urea (pH 7.4); then broken by sonicator with solution containing 200 µg/mL lysozyme. The lysate was purified by Chelating Sepharose Fast Flow (GE Healthcare, Illinois, USA). The protein samples were analyzed by 12% sodium dodecyl sulfate polyacrylamide gel electrophoresis (SDS-PAGE) and determined by staining with Coomassie brilliant blue, as previously described^22^. Proteins were purified and condensed with the dialysis membrane, then aliquoted and kept at − 20 °C until used to generate antibodies.

### Production of rabbit anti-EEHV DNAPol antibodies

Rabbit polyclonal antibodies against EEHV DNAPol proteins were generated and obtained, as previously described^22^, with minor modification. Briefly, purified EEHV DNAPol proteins, including DNAPolF1E1, DNAPolF2E1 and DNAPolF1E4, at concentrations of 0.5 mg/mL, were mixed with an equal volume of Montanide incomplete seppic adjuvant (Seppic, Paris, France), then injected subcutaneously into the New Zealand White rabbits (2 animals/fragment) five times each, at 2-week intervals. One animal served as the negative control. Prior to each injection, blood was sampled and an enzyme-linked immunosorbent assay (ELISA) was performed, as previously described, to determine antibody titer^22^. Two weeks after the last immunization, animals were sacrificed, and antisera were obtained. Antibodies were purified by HiTrap Protein A HP antibody purification column (GE Healthcare, Illinois, USA) and used in subsequent studies.

### SDS-PAGE and western immunoblotting

SDS-PAGE and western immunoblotting were performed according to a previously published protocol^22^. Briefly, EEHV DNAPol recombinant proteins or tissue lysate of the EEHV1A-HD calf were mixed with an equal volume of 2× Laemmli buffer, loaded in 10–12% SDS gel, and subjected to electrophoresis at 130 V for 90 min. Proteins were transferred from the SDS-PAGE gel to the nitrocellulose membrane through a semi-dry electrophoretic transfer cell for 1 h, and subjected to immunoblot analysis. The blotted membranes were poured into 5% skim milk for 30 min, and then soaked with rabbit anti-EEHV DNAPol (1:2,500) at RT for 2 h with agitation. Then, the membranes were washed three times with PBS before incubating with biotinylated goat anti-rabbit IgG secondary antibodies (1:5,000; EMD Millipore, Billerica, MA) in PBS/0.05% Tween-20 for 45 min at RT. Subsequently, they were washed and incubated with avidin–biotin peroxidase complex (ABC) reagent (Thermo Scientific) for 30 min at RT. The membranes were developed with 3,3′-diaminobenzidine tetrahydrochloride (DAB) substrate and the signals were observed. For further characterization of EEHV-infection, anti-EEHV DNAPol antibodies of fragments that showed a strong positive signal by western immunoblotting were then selected for further immunocytological investigation.

### Immunohistochemistry

Immunohistochemistry followed the ABC method, as described previously^41^. Briefly, FFPE blocks of EEHV-HD and EEHV-negative control were cut into 3-µm thick sections. Then, they were dewaxed, rehydrated and the antigens retrieved by boiling in a microwave for 15 min with citrate buffer pH 6.0. Endogenous peroxidase activity and non-specific binding in the tissues was blocked by 3% H_2_O_2_ in methanol and 2.5% bovine serum albumin (BSA), respectively. Then, they were incubated with rabbit anti-EEHV DNAPol (1:600) at 37 °C for 2 h. After washing three times with PBS, biotin conjugated goat anti-rabbit secondary antibody (1:200) was added, followed by ABC (Thermo Scientific, USA) for 1 h at RT. Antibody binding was visualized using DAB for 5 min at RT, followed by washing with tap water; nuclei were counterstained by hematoxylin. For negative control, normal rabbit serum was used instead of primary antibodies. Slides were observed and photos were taken under a light microscope.

### Double immunofluorescence

The EEHV-HD and EEHV-negative elephant tissues were double immunofluorescent stained using 3-µm thick section slides, as previously described^22^. Briefly, after deparafinization, sections were treated with 0.25% Triton X-100 in PBS (0.25% PBST) for 15 min. Then, they were incubated with 1% BSA in PBST for 30 min at RT, followed by incubation with specific primary antibodies diluted with 1% BSA in 0.25% PBST at 37 °C for 2 h. The primary antibodies used in this study were rabbit polyclonal anti-EEHV DNAPol and mouse monoclonal anti-Iba-1 (1:400; Millipore Corporation, Billerica, MA, USA). After washing three times with PBS, the secondary antibodies were incubated for 45 min at RT with Cy3-conjugated goat anti-mouse and FITC-conjugated goat anti-rabbit antibodies (all from Jackson ImmunoResearch, Suffolk, UK), at a dilution of 1:200. The nuclei were counterstained and slides were analysed under an inverted fluorescent microscope.

### Data analysis

Positive cells were determined by double immunolabeling, as previously described^42^. All data were included in a descriptive analysis using GraphPad Prism 5 (GraphPad Inc., La Jolla, CA, USA).

## Supplementary information

Supplementary Information.

## Data Availability

All data generated or analyzed during this study are included in this published article, and its Supplementary Information files.
