# Supplementary material for: In vivo characterization of target cells for acute elephant endotheliotropic herpesvirus (EEHV) infection in Asian elephants (Elephas maximus)
Source: Sci Rep. 2020 Jul 9;10:11402. doi: 10.1038/s41598-020-68413-4 (PMC7347588; doi:10.1038/s41598-020-68413-4)
Supplement: Supplementary file 1 — Supplementary Information. [file 41598_2020_68413_MOESM1_ESM.docx]

***In vivo* characterization of target cells for acute elephant endotheliotropic herpesvirus (EEHV) infection in Asian elephants (*Elephas maximus*)**

Thunyamas Guntawang^1^, Tidaratt Sittisak^1^, Saralee Srivorakul^1,2^, Varankpicha Kochagul^2^, Kornravee Photichai^1,2^, Chatchote Thitaram^3,4^, Nattawooti Sthitmatee^1^, Wei-Li Hsu^5^, Kidsadagon Pringproa^1,3 *^

^1^ Department of Veterinary Biosciences and Veterinary Public Health, Faculty of Veterinary Medicine, Chiang Mai University, Chiang Mai 50100, Thailand

^2^ Veterinary Diagnostic Laboratory, Faculty of Veterinary Medicine, Chiang Mai University, Chiang Mai 50100, Thailand

^3^ Center of Excellence in Elephant and Wildlife Research, Chiang Mai University, Chiang Mai 50100, Thailand

^4^ Department of Companion Animals and Wildlife Clinics, Faculty of Veterinary Medicine, Chiang Mai University, Chiang Mai 50100, Thailand

^5^ Graduate Institute of Microbiology and Public Health, College of Veterinary Medicine, National Chung Hsing University, Taichung 402, Taiwan

^*^ Corresponding author

E-mail: [kidsadagon.p@cmu.ac.th](mailto:kidsadagon.p@cmu.ac.th) (KP)

**Supplementary information**

**S1 Figure.** Western blot analysis of the EEHV DNAPolF2E1 recombinant protein

**

**

The EEHV DNAPolF2E1 recombinant protein, which was used as immunogen in the present study, was SDS-PAGE and immunoblot with the rabbit anti-EEHV DNAPolF2E1 antibodies. The proteins size of ~31.2 kDa (arrow), which indicated the molecular weight size of DNAPolF2E1, was observed. Lane M = protein molecular weight marker. Image was modified with Adobe Photoshop CS6 v.13.0.1.

**S2 Figure.** Western blot analysis demonstrating the EEHV DNA polymerase antigen from the tissue lysate of the EEHV1A-HD calf





Mixed tissue lysate of the heart, lung, spleen and kidney of the EEHV1A-HD calf was subjected to SDS-PAGE and immunoblot with the rabbit anti-EEHV DNAPolF2E1 antibodies. The proteins size of ~117 kDa (arrow), which indicated the molecular weight size of EEHV DNA polymerase protein, was observed. Lane M = protein molecular weight marker. Image was modified with Adobe Photoshop CS6 v.13.0.1.

**S3 Figure.** Representative photomicrographs of the EEHV-negative elephant tissues immunolabeling with the rabbit anti-EEHV DNAPolF2E1 antibodies


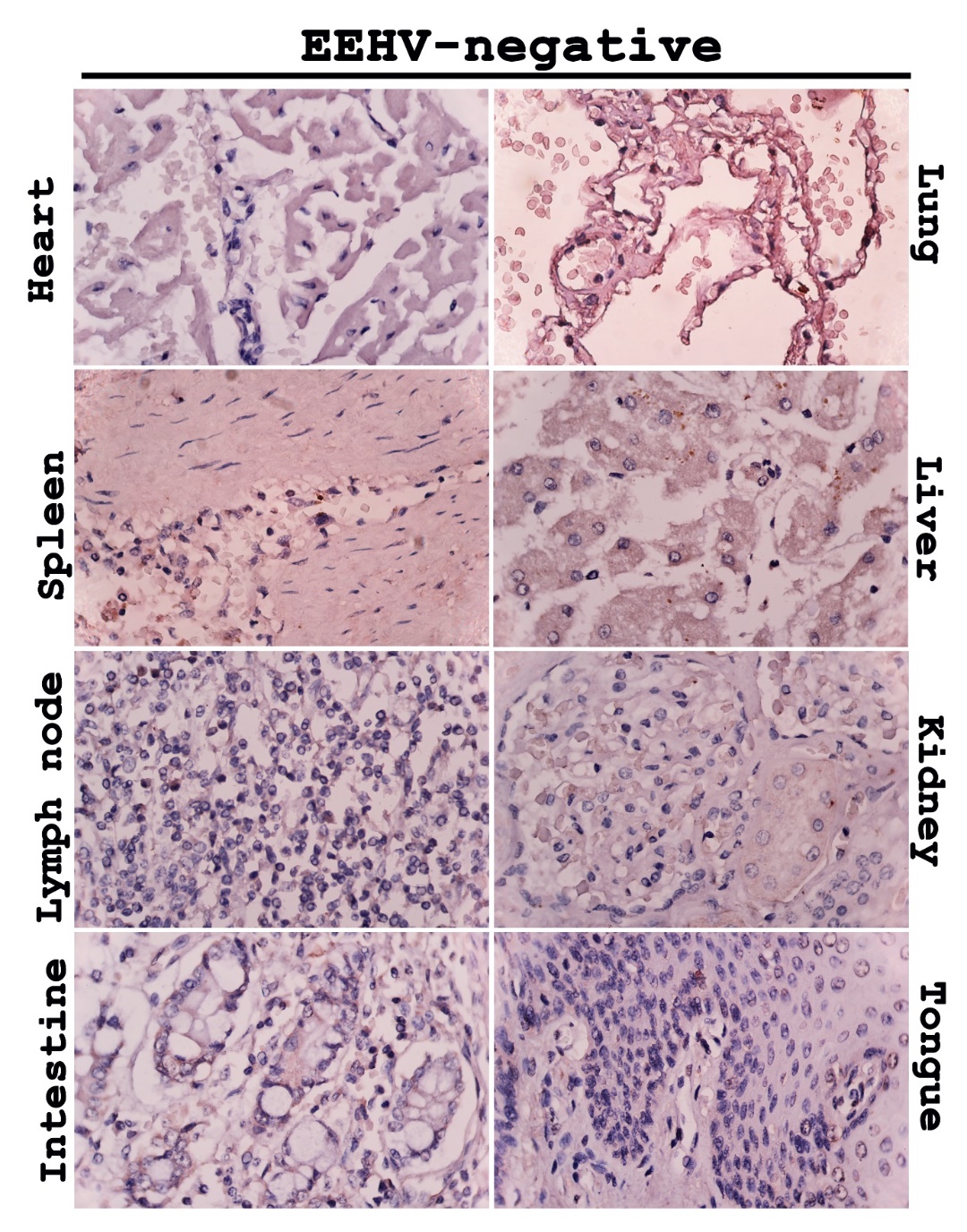


Elephant tissues that died due to non-EEHV related causes were immunohistochemical labeling with the rabbit anti-EEHV DNAPolF2E1 antibodies. The results showed little or no staining in the heart, lung, spleen, liver, lymph node, kidney, intestine and tongue. Image was modified with Adobe Photoshop CS6 v.13.0.1.

**S4 Figure.** Representative photomicrographs of the EEHV1A- and EEHV4-HD tissues immunolabeling with the rabbit pre-immunized sera


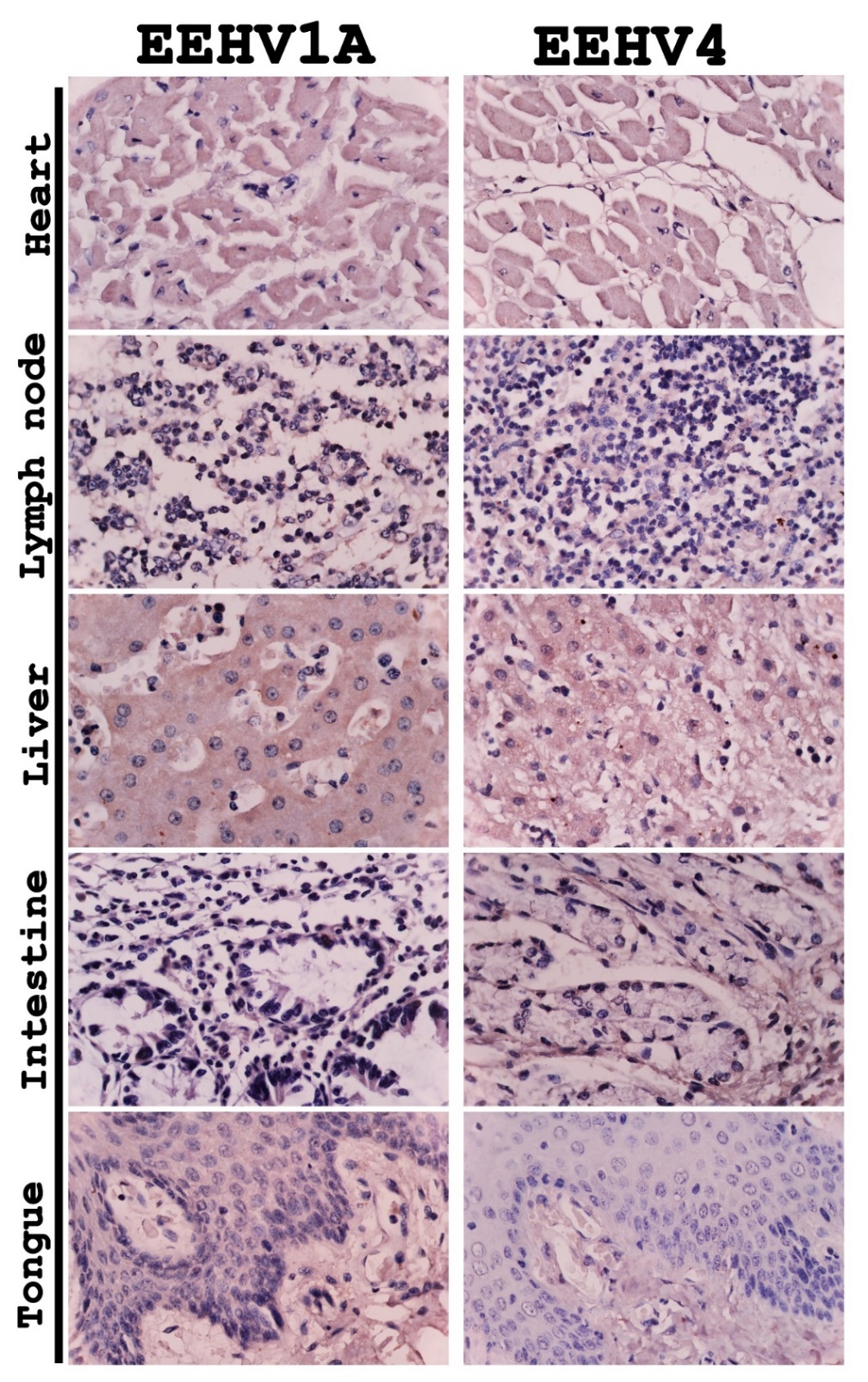


Formalin-fixed, paraffin embedded tissues (FFPE) of the EEHV1A- and EEHV4-HD calves were subjected to immunohistochemical labeling with the rabbit pre-immunized sera, as primary antibody. The results showed little or no staining in the tested organs, including the heart, lymph node, liver, kidney, intestine and tongue. Image was modified with Adobe Photoshop CS6 v.13.0.1.
